# Feasibility of measuring spleen stiffness with MR elastography and splenic volume to predict hepatic fibrosis stage

**DOI:** 10.1371/journal.pone.0217876

**Published:** 2019-05-31

**Authors:** Yi-Wen Cheng, Ya-Chien Chang, Yao-Li Chen, Ran-Chou Chen, Chen-Te Chou

**Affiliations:** 1 Department of Biomedical Imaging and Radiological Sciences, National Yang-Ming Medical University, Taipei City, Taiwan; 2 Department of Radiology, Changhua Christian Hospital, Changhua City, Taiwan; 3 Transplant Medicine & Surgery Research Centre, Changhua Christian Hospital, Changhua City, Taiwan; 4 School of Medicine, Kaohsiung Medical University, Kaohsiung City, Taiwan; 5 Department of Molecular Biotechnology, College of Biotechnology and Bioresources, Dayeh University, Changhua City, Taiwan; Taipei Veterans General Hospital, TAIWAN

## Abstract

**Aim:**

The aim of this study was to investigate the relationship between spleen stiffness value, splenic volume and the liver fibrosis stages.

**Materials and methods:**

This retrospective study was approved by the institutional review board of our institute. We enrolled 109 patients that had undergone abdominal MR imaging and histopathological examination. The preoperative MR imaging, MR elastography and laboratory data were reviewed. Liver stiffness and spleen stiffness were determined with MR elastography, and splenic volume was calculated. Liver fibrosis stage was determined using surgical pathology.

**Results:**

The correlation coefficient between the liver stiffness and the fibrosis stage was r = 0.72 and r = 0.62 when the passive driver was on right chest wall and the left chest wall, respectively. The correlation coefficient between the spleen stiffness and the fibrosis stage was r = 0.63 and r = 0.18 when the passive driver was on the left chest wall and the right chest wall, respectively. The correlation coefficient between the splenic volume and the fibrosis stage was r = 0.31. The diagnostic performance of spleen stiffness was similar to liver stiffness in prediction of advanced liver fibrosis. The combination of spleen stiffness and liver stiffness provided greater sensitivity in prediction of advanced fibrosis than spleen or liver stiffness alone, but no significant difference was found.

**Conclusion:**

According to our study, the spleen stiffness value was useful in staging liver fibrosis. The combination of spleen stiffness and liver stiffness could provide higher diagnostic sensitivity than liver stiffness alone in prediction of advanced fibrosis.

## Introduction

Chronic liver disease is a major health concern and can lead to liver fibrosis. Liver fibrosis can be reversible with specific treatment; however, treatment is associated with an increased risk of morbidity and mortality [1,2]. Thus, it is essential to detect hepatic fibrosis in advance. Currently, liver biopsy has been used as the gold standard to detect hepatic fibrosis. However, it has several limitations such as a lower patient acceptance, sampling error and inter-observer interpretation variation [3]. There is a clinical demand for a noninvasive and sensitive method to assess liver fibrosis. MR elastography (MRE), using an MRI-based quantitative shear wave elastography method, has been shown to be an accurate method for staging liver fibrosis with advantages of large organ coverage, excellent inter-scan reproducibility and inter-reader agreement [4–7]. Because of the high accuracy of MRE in hepatic fibrosis staging, MRE could potentially replace invasive liver biopsy for fibrosis staging [8,9]. Using MRE in determination of hepatic stiffness and splenic stiffness might provide a comprehensive assessment of liver fibrosis and portal hypertension [10–12]. It has been reported that splenic stiffness measurement with MRE is also useful for predicting clinical complications in cirrhosis patients [13–15].

Splenomegaly is very common in patients with advanced liver fibrosis, and splenic volume increases with the degree of fibrosis stage [16,17]. Talwalkar et al. also reported a strong relationship between liver stiffness and splenic stiffness [18]. However, the relationship between splenic volume, hepatic fibrosis, and splenic stiffness remains poorly understood. We hypothesized that spleen stiffness might have an association with hepatic fibrosis in that spleen stiffness increases with the development of fibrosis. Therefore, the purpose of our study was to investigate the relationship between spleen stiffness value, splenic volume and the liver fibrosis stages.

## Materials and methods

### Patients

**Fig 1:**
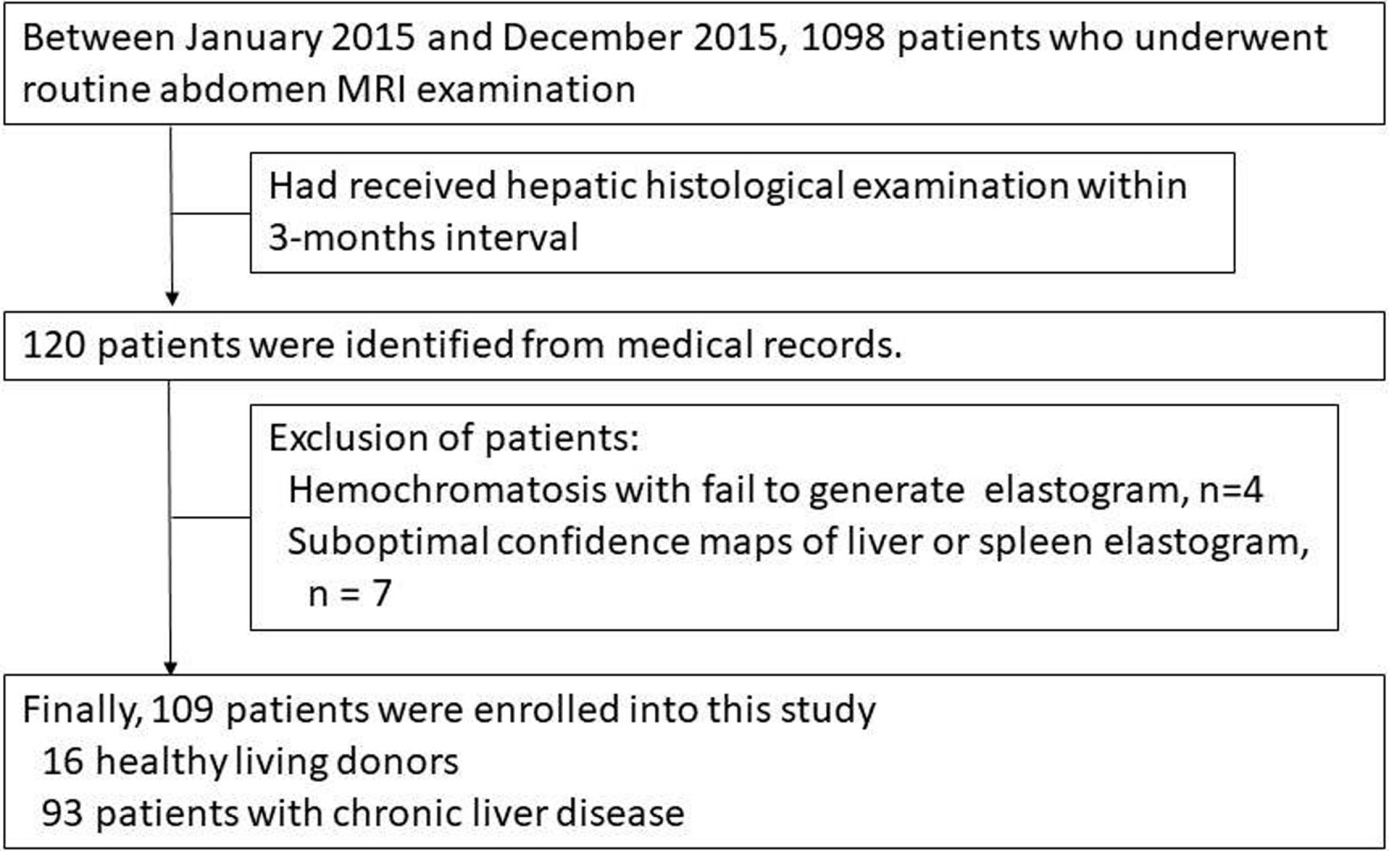
Flowchart of patients enrolled in our study.

The institutional review board of our institute approved this retrospective study, and the informed consent was waived. Between January 2015 and December 2015, patients having undergone abdominal MR imaging and histopathological examination within a 3-month interval were retrospectively identified from medical records (Fig 1). Finally, 109 patients were enrolled into this study. The study’s patient characteristics were demonstrated in Table 1. There were 17 patients with fibrosis stage 0 (F0), 15 with fibrosis stage 1 (F1), 23 with fibrosis stage 2 (F2), 21 with fibrosis stage 3 (F3), and 33 with fibrosis stage 4 (F4).

**Table 1:** The patient characteristics of the study population.

	Healthy living donor n = 16(%)	Patients with chronic liver disease n = 93(%)
Age (years)	32.06±6.55	62.46±11.39
Gender		
Male	8(50.0)	72(77.4)
Female	8(50.0)	21(22.6)
Underlying liver disease		
HBV		47(50.5)
HCV		28(30.1)
HBV+HCV		4(4.3)
Alcoholism		3(3.2)
NASH		2(2.2)
Others		9(9.7)
Histological fibrosis stage		
F0	16(100)	1(1.1)
F1		15(16.1)
F2		23(24.7)
F3		21(22.6)
F4		33(35.5)

HBV, Hepatitis B virus; HCV, Hepatitis C virus; NASH, non-alcoholic steatohepatitis; F0-4, Fibrosis stage 0–4.

### Imaging process

All MR imaging was performed via a 1.5T MR scanner (Aera, Siemens, Erlangen, Germany) with a 16-channel body coil. The MR elastography used an active driver system (Resoundant, Rochester, MN, USA) to generate acoustic vibrations. A 19 cm diameter cylindrical passive driver was placed against the right chest wall and the left chest wall with the center of the passive driver at the level of the xiphoid process. The passive driver was connected by a flexible plastic tube to the acoustic active driver outside the MRI room. Acoustic vibrations at 60 Hz were generated by the active driver to detect shear waves propagating in the liver and spleen. An MRE pulse sequence with an axial 2D gradient echo was used to detect the propagating shear waves. The parameters of the MRE pulse sequence were the following: imaging frequency, 63.5 MHz; section thickness, 5 mm; acquisition matrix, 256 × 64; FOV, 40 cm × 40 cm; TR/TE, 50/22.7; bandwidth, 260 Hz/pixel; and flip angle, 25°. The scanning time was 21 seconds for one imaging slice. An MRE image was obtained with breath-hold at the end-expiratory period for each image slice. Five axial slices for the liver (passive driver on the right chest wall) and three axial slices for the spleen (passive driver on the left chest wall) in different levels were obtained for each patient. The imaging slices for each patient were arranged so that the center slice was located at the largest organ diameter on coronal scout view and each slice gap was 1cm. The elastograms were generated automatically using intrinsic postprocessing software. In measurement of the liver/spleen stiffness, the values were depicted in kilopascals. Confidence maps of the elastogram were generated automatically to indicate the regions with adequate wave amplitudes for measurement. The confidence mapping of the elastogram was then checked by the intrinsic software for imaging quality assessment. In case a suboptimal elastogram was found, changing the position of the passive driver on the chest wall to improve the elastogram was done in our daily practice.

### Imaging analysis

**Fig 2:**
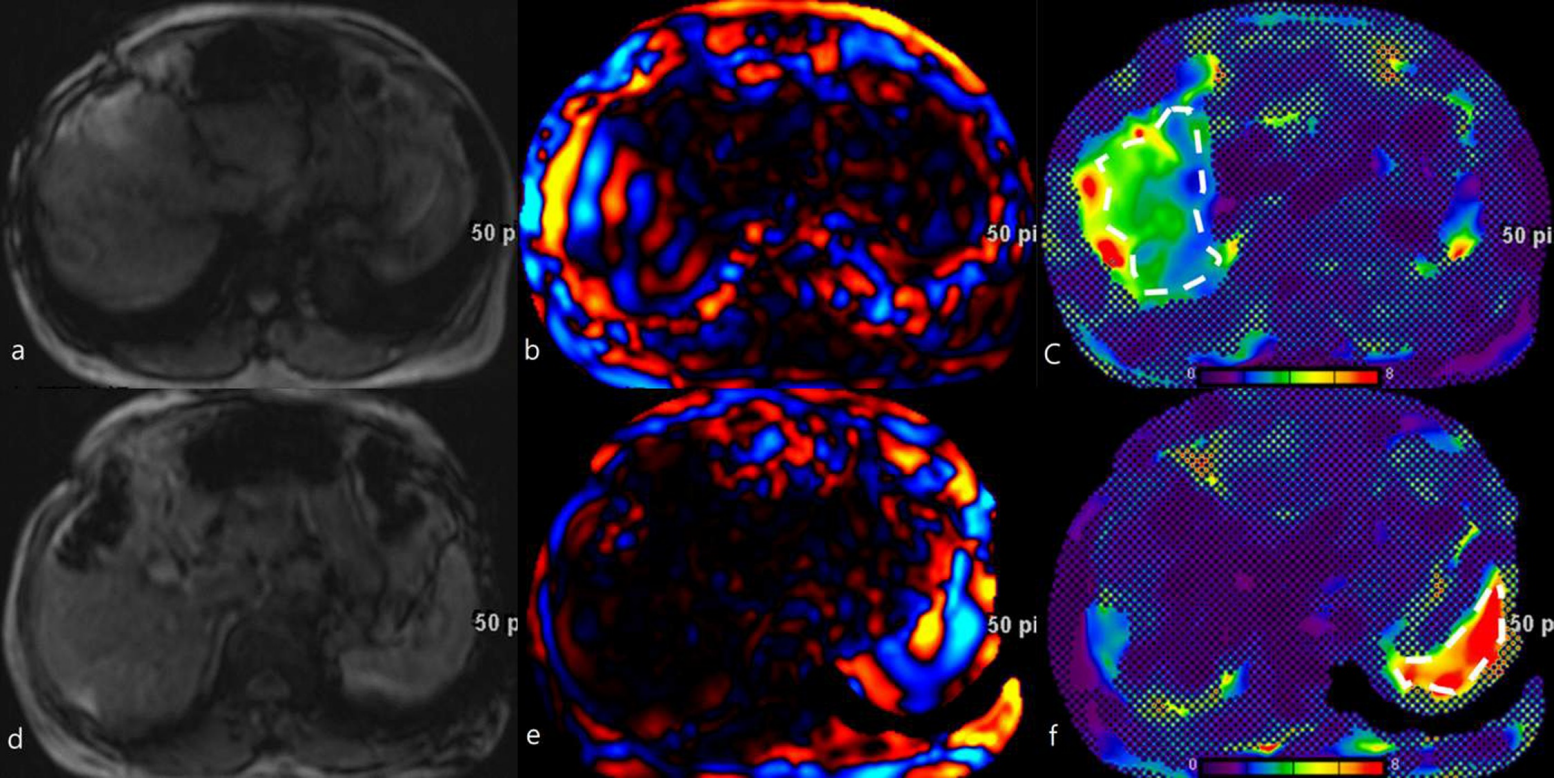
A 53-year-old male with chronic hepatitis B infection and pathologic fibrosis stage 3. While the passive driver was on the right chest wall: (a) MR magnitude images provided anatomic information, (b) Wave images from the MRE acquisition at 60 Hz showing propagating shear waves, (c) Region-of-interest (white-dashed line) for liver stiffness measurement was drawn on an elastogram with confidence mapping. The liver stiffness was 3.17 kPa. While the passive driver was on the left chest wall: (d) MR magnitude images provided anatomic information, (e) Wave images from the MRE acquisition at 60 Hz showing propagating shear waves, (f) Region-of-interest (white-dashed line) for spleen stiffness measurement was drawn on an elastogram with confidence mapping. The spleen stiffness was 6.63 kPa.

Imaging review and analysis were performed using a dual screen diagnostic workstation (GE Healthcare, Milwaukee, WI, USA). A radiologist evaluated the MR elastography images, including anatomic images, the MRE elastogram, and the propagating wave images. The radiologist had more than 20 years of clinical experience in abdominal MRI and was blinded to the pathological finding. The propagating wave images should be checked first for imaging quality in measuring liver/spleen stiffness. The region of interest (ROI) was manually drawn to include only the parenchyma of the liver or spleen as large as possible in each image with confidence mapping (Fig 2). The ROI areas should be free of reflections and interference patterns, and wave propagations among ROI areas were regular. The ROI areas should avoid the hepatic/splenic capsule, large vascular structures and tumors. The shear stiffness value of the tissue was determined on an elastogram using a confidence map. If the ROI contained less than 1000 pixels, the image quality was considered suboptimal and the slice was excluded from further analysis. The overall mean stiffness value for each elastography image was recorded.

### Splenic volume measurement

Based on 3D FFE T1W images (TR,10.2–10.7 ms; TE, 5 ms; matrix, 192 x 256; FOV, 38–40 cm; slice thickness, 5 mm; flip angle, 15°), the splenic volume was calculated by manually drawing the spleen contour on every slice and recording the measurements of the area (mm^2^). The calculation of splenic volume (cm^3^) was obtained by adding up all the measurements of the spleen areas, multiplying by 5mm and then dividing by 1000.

### Pathological analysis

The reference of standard for hepatic fibrosis stage was based on the results of pathological examination. Liver samples were obtained from 93 patients who underwent surgery (n = 80) or percutaneous liver biopsy (n = 13) and from 16 liver donors. The specimens of all patients were stained with H&E and Masson trichrome. All pathological specimens were interpreted by a pathologist who was blinded to the clinical data and MRE data. The METAVIR system was used to classify the hepatic fibrosis stage. There were five grades (F0-F4) in the METAVIR system. According to the METAVIR system, stage F0 was defined as no fibrosis, stage F1 was defined as portal fibrosis without septa, stage F2 was defined as portal fibrosis with a few septa, stage F3 was defined as numerous septa without cirrhosis, and F4 was defined as cirrhosis. A fibrotic stage of F3 or above was considered to be advanced liver fibrosis.

### Statistical analysis

Spearman rank correlation was used to test the association between MRE liver stiffness value, spleen stiffness value, splenic volume and hepatic pathological fibrosis stage. Chi-square test was used to compare the differences of fibrosis stages between HBV and non-HBV groups. The differences between the means of the liver stiffness and spleen stiffness values at each fibrosis stage were compared using a one-way analysis of variance (ANOVA). A receiver operating characteristic (ROC) curve analysis was applied to detect the optimal cut-off points and area under the ROC curve (AUROC) for liver stiffness, spleen stiffness, and splenic volume. The AUROC values were compared by DeLong’s test. The highest Youden index (the sum of sensitivity and specificity) was used in determining the optimal cutoff values for liver stiffness, spleen stiffness and splenic volume. Statistical significance was set at a p-value less than 0.05, and all tests were two-tailed. All statistical analyses were performed using SPSS software (version 16.0, SPSS Inc., Chicago) and MedCalc software (version 13.1.2, Mariakerke, Belgium).

## Results

### Liver stiffness measurements

Table 2 shows the liver stiffness values of the right hepatic lobe that were obtained via placement of the passive driver on the right chest wall and on the left chest wall. In liver stiffness measurements of the right hepatic lobe, no patient had a suboptimal elastogram when the passive driver was on the right chest wall, but 5 patients (F0, 2 patients; F3, 1; F4, 2) had suboptimal elastograms when the passive driver was on the left chest wall. We divided our patients into chronic hepatitis B (CHB) and non-CHB subgroups. The mean liver stiffness value of stage F4 patients with CHB was significantly lower than that of stage F4 patients without CHB (Table 2).

**Table 2:** The liver stiffness and spleen stiffness values at each fibrosis stage.

		F0	F1	F2	F3	F4	*p* value
Total (N = 109)							
	Liver stiffness (R’t)(kPa)	2.13±0.39	2.92±0.61	3.02±0.43	3.70±0.93	5.14±2.66	<0.001
	Liver stiffness (L’t)(kPa)	2.14±0.34	3.05±0.82	2.93±0.47	3.44±0.82	4.42±1.58	<0.001
	Spleen stiffness (R’t)(kPa)	4.00±0.99	3.79±.083	3.91±1.61	4.15±1.04	4.63±1.87	0.284
	Spleen stiffness (L’t)(kPa)	4.41±0.46	5.03±0.87	4.57±1.24	5.85±1.31	6.77±1.49	<0.001
HBV; n = 47(%)		0(0)	6 (12.8)	16(34.0)	9(19.1)	16(34.0)	0.001^a^
	Liver stiffness (R’t)(kPa)	-	2.84±0.38	3.02±0.44	3.63±1.08	3.70±1.20	0.097
	Liver stiffness (L’t)(kPa)	-	2.97±1.01	3.00±0.49	3.39±0.78	3.69±1.47	0.275
	Spleen stiffness (R’t)(kPa)		3.33±1.04	3.95±1.51	4.16±0.98	4.61±1.66	0.286
	Spleen stiffness (L’t)(kPa)		4.76±0.82	4.67±1.19	6.14±1.68	6.13±1.12	0.003
Non-HBV; n = 58(%)		17(29.3)	7(12.1)	7(12.1)	11(19.0)	16(27.6)	
	Liver stiffness (R’t)(kPa)	2.13±0.39	2.77±0.50	3.01±0.44	3.76±0.88	6.67±2.96	<0.001
	Liver stiffness (L’t)(kPa)	2.14±0.34	2.91±0.59	2.76±0.39	3.41±0.91	5.21±1.38	<0.001
	Spleen stiffness (R’t)(kPa)	4.00±0.99	4.02±0.51	3.80±2.00	4.16±1.20	4.64±2.16	0.752
	Spleen stiffness (L’t)(kPa)	4.41±0.46	4.98±0.79	4.36±1.41	5.61±1.02	7.50±1.53	<0.001

R’t, passive driver placed on right chest wall; L’t, passive driver placed on left chest wall; F0-4, Fibrosis stage 0–4; Values are depicted as mean ±standard deviation.

^a^ The differences of fibrosis stages between HBV and non-HBV groups were compared using chi-square test.

**Fig 3:**
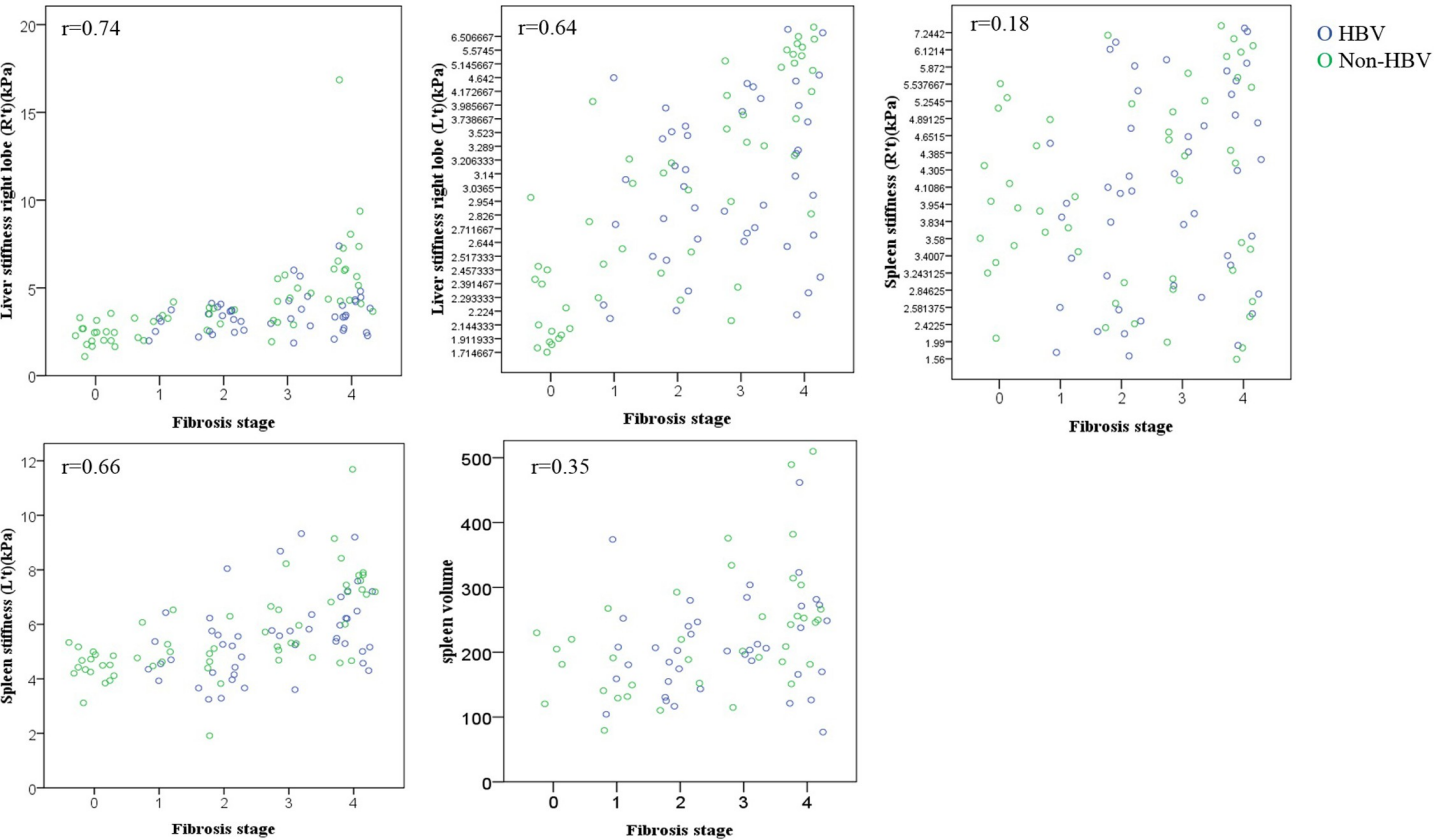
Scatterplot with jittered points illustrating the relationship between liver/spleen stiffness, splenic volume and fibrosis stage.

According to Spearman’s rank correlation, the correlation coefficient between the liver stiffness and the fibrosis stage was r = 0.72 when the passive driver was on the right chest wall and r = 0.62 when the passive driver was on the left chest wall (Table 3). Scatterplot with jittered points to display the relationship between the liver stiffness and the fibrosis stage in two groups was presented in Fig 3. In our result, placing the passive driver near the right hepatic lobe led to a higher success rate and a higher diagnostic performance than placing the passive driver far from the right hepatic lobe. Thus, the passive driver should be placed on the right chest wall when measuring liver stiffness of the right hepatic lobe.

**Table 3:** Correlation coefficients between liver/spleen stiffness, splenic volume and fibrosis stage.

	Liver stiffness (R’t)(kPa)	Liver stiffness (L’t)(kPa)	Spleen stiffness (R’t)(kPa)	Spleen stiffness (L’t)(kPa)	Splenic volume
Fibrosis (F0-4)	0.72**	0.62**	0.18	0.63**	0.31**
Liver stiffness (R’t)(kPa)		0.90**	0.08	0.62**	0.34**
Liver stiffness (L’t)(kPa)			0.16	0.61**	0.24*
Spleen stiffness (R’t)(kPa)				0.29**	-0.07
Spleen stiffness (L’t)(kPa)					0.41**

R’t, passive driver placed on right chest wall; L’t, passive driver placed on left chest wall; F0-4, Fibrosis stage 0–4;

**Correlation is significant at the 0.01 level (2-tailed);

*Correlation is significant at the 0.05 level (2-tailed).

#### Spleen stiffness measurements

A comparison of the spleen stiffness values acquired when the passive driver was placed at the right location and the left location is shown in Table 2. In spleen stiffness measurements, no patient had a suboptimal elastogram when the passive driver was on the left chest wall, but 7 patients (F0, 5 patients; F2, 1; F3, 1) had suboptimal elastograms when the passive driver was on the right chest wall. Using Spearman’s rank correlation, the correlation coefficient between the spleen stiffness and the fibrosis stage was r = 0.63 when the passive driver was on the left chest wall, but it was only r = 0.18 when the passive driver was on the right chest wall (Table 3). According to our result, the location of the passive driver being near the spleen led to a higher diagnostic performance than when the location was far from the spleen. For spleen stiffness measurements, the passive driver should be placed on the left chest wall.

### Splenic volume measurement

**Fig 4:**
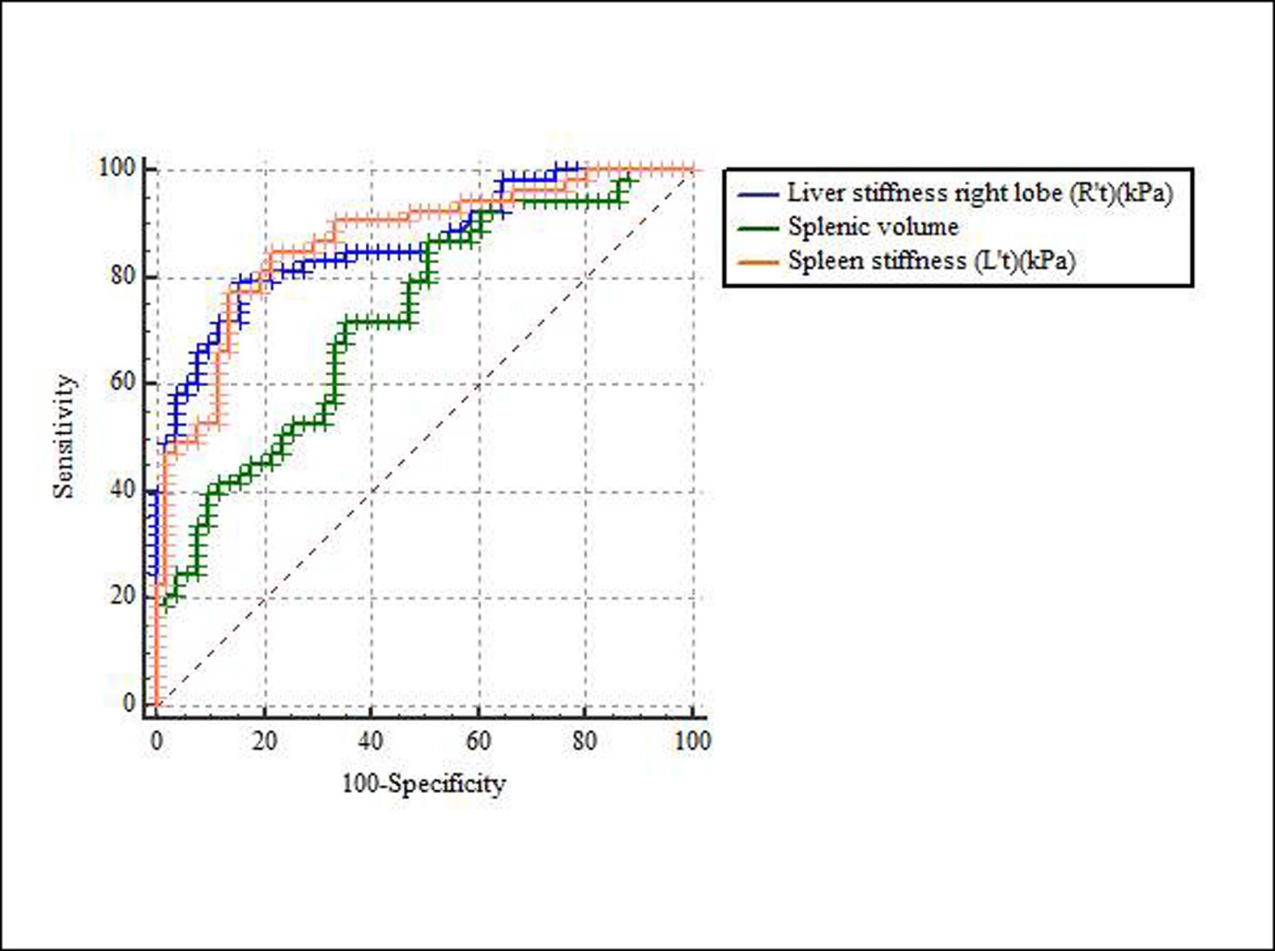
Receiver operating characteristic (ROC) curve for predicting advanced liver fibrosis (F3-F4).

The mean splenic volume data (mean±standard deviation) at each fibrosis stage are as follows: F0, 197.29±73.61 cm^3^ (range, 82.76~337.63 cm^3^); F1, 198.36±87.19 cm^3^ (79.13~398.6 cm^3^); F2, 186.14±66.08 cm^3^ (89.39~332.23 cm^3^); F3, 245.06±81.81 cm^3^ (170.31~520.12 cm^3^); F4, 286.15±139.94 cm^3^ (111.26~706.74 cm^3^). The correlation coefficient between the splenic volume and the fibrosis stage was r = 0.31 according to Spearman’s rank correlation. The patients were further divided into two subgroups: a group of 55 patients whose conditions ranged from no fibrosis to substantial fibrosis (F0~F2) and a group of 54 patients with advanced liver fibrosis (F3~F4). Table 4 contains the diagnostic performances, based on ROC analysis, of the following measurements in regard to predicting advanced liver fibrosis: liver stiffness value, spleen stiffness value, splenic volume and the combination of liver stiffness and spleen stiffness values. The spleen stiffness values and the liver stiffness values showed similar area under curve values (AUC, 0.86) in prediction of advanced liver fibrosis (Fig 4). The combination of spleen stiffness and liver stiffness provided greater AUC (0.90) than spleen or liver stiffness alone, but no significant difference was found.

**Table 4:** The statistical results of spleen stiffness, spleen volume, liver stiffness and the combination of spleen stiffness and liver stiffness for predicting advanced liver fibrosis (F3-F4).

	Spleen stiffness (L’t) (kPa)	Splenic volume (cm^3^)	Liver stiffness (R’t) (kPa)	Liver stiffness+ spleen stiffness
Cut-off value	5.00	200.54	3.20	
AUC (95% CI)	0.86 0.78 to 0.92	0.70 0.61 to 0.79	0.86 0.78 to 0.92	0.90 0.82–0.95
Sensitivity (%)	85.19	72.22	79.25	96.23
Specificity (%)	76.36	61.82	84.31	70.59

R’t, passive driver placed on right chest wall; L’t, passive driver placed on left chest wall; CI, confidence interval; AUC, area under curve.

## Discussion

In our study, the combination of spleen stiffness and liver stiffness could provide higher AUC than spleen or liver stiffness alone, although no significant difference was found. There were two major components for the elevation of hepatic stiffness in patients with chronic liver disease: structural change or fibrosis and portal/venous pressure [19]. Talwalkar et al. reported a strong linear relationship between liver stiffness and splenic stiffness (r^2^ = 0.75), and a splenic stiffness value ≥ 10.5 kPa was associated with a 100% rate of esophageal varices [18]. The measurement of spleen stiffness might reflect the portal pressure status [20]. Yin et al. also reported a strong correlation (r > 0.80) between splenic stiffness measured with MRE and hepatic vein-portal vein gradient [21]. The combination of spleen stiffness and liver stiffness might provide a higher diagnostic performance in detecting advanced fibrosis. However, limited data of splenic stiffness values measured by MRE is available to date for discrimination of different fibrosis stages and further study with a large patient group is needed.

### Liver fibrosis of MRE

In our study, MRE showed good performance in determining the liver fibrosis stage, and liver stiffness depicted good correlation (r = 0.72) with the histopathologic liver fibrosis stage. Our result was similar to several previous studies of patients with chronic hepatitis B/C or nonalcoholic fatty liver disease [22–24]. Meanwhile, the mean liver stiffness values of stage F4 patients with CHB was significantly lower than that of the stage F4 patients without CHB. Similar results were also found in previous ultrasound-based elastography [25] and MRE [26] studies. The phenomenon might be due to that chronic hepatitis B patients tended to be macronodular, have heterogeneous fibrosis of liver and have less fibrotic tissue than chronic hepatitis C patients [27,28]. It should be kept in mind that different underlying etiologies of chronic liver disease might account for the differences in the cutoff values for fibrosis staging.

As shown in Table 2, the splenic stiffness measurements were significantly different based on whether the passive driver was placed on the right chest wall or the left chest wall. The passive driver being on the left chest wall provided higher correlation between splenic stiffness and liver fibrosis stage compared to the passive driver being on the right chest wall. Our result was similar to Mannelli’s study that reported the mean splenic stiffness (3.56 ± 0.59 kPa) of healthy volunteers with the driver placed on the right side of the abdomen was significantly different than the mean splenic stiffness (4.26 ± 0.63 kPa) with the driver on the left side [29]. One reason might be that the wave amplitude attenuates as the distance from the passive driver increases. Using the right chest position, a propagated wave passes through the liver and other visceral structures before reaching the spleen. On the other hand, in the left chest location the vibration reaches the spleen more directly and should provide a sufficient shear wave. Another reason might be that there are many organ interfaces on the path of the wave propagation; these may cause wave reflection and interference and further affect the properties of the initial wave [30]. In stiffness measurements of the right hepatic lobe with MR elastography during our study, there was also a significant difference between placing the passive driver on the right chest wall and placing it on the left chest wall. The passive driver being on the right chest wall provided higher correlation between hepatic stiffness value and hepatic fibrosis stage than the passive driver being on the left chest wall. According to our results, when determining liver/splenic stiffness with MR elastography, the passive driver should be placed on the nearest chest wall of the target organ.

Our study revealed that splenic volume has a positive correlation with liver fibrosis stage and can be used to estimate the degree of fibrosis. Splenic volume had been reported to be useful in monitoring and diagnosing liver fibrosis stage and liver cirrhosis [17,31]. However, the increase in splenic volume was only observed for patients with advanced fibrosis. Using a cutoff value of 198.47 cm^3^ for splenic volume, we could differentiate advanced liver fibrosis (F3-F4) from relatively early fibrosis stages (F0-F2) with a sensitivity of 72.22% and a specificity of 61.82%. Liu et al. reported splenic volume with a cutoff value of 358.67 cm^3^ was useful in differentiating advanced liver fibrosis, but not in early fibrosis stage [31]. According to our results, splenic volume was insensitive to prediction of early fibrosis stages, however, it was a good marker for advanced stages.

There are some potential limitations in the study. First, this study was a retrospective study with a relatively small patient number. In the study population, the underlying disease and the distribution of fibrosis stages were uneven. Second, the influences of liver steatosis, iron deposition and other confounder factors for MRE measurement were not evaluated in this study. Third, 2D gradient echo MRE was used in our study. 3D MRE enables the acquisition of the full wave field, additional parameters, artifact reduction and fewer assumptions about the material model during inversion [32]. However, a comparison of the diagnostic accuracies of 2D and 3D MRE reported they were similar [33].

## Conclusions

Our study verified the feasibility of staging liver fibrosis via splenic volume calculation and MR elastography of the spleen. Spleen stiffness could provide useful information in characterization of different liver fibrosis stages. A combination of spleen stiffness and liver stiffness could provide higher sensitivity in detection of advanced fibrosis stages than liver stiffness alone.
